# Visual mismatch negativity to vanishing parts of objects in younger and older adults

**DOI:** 10.1371/journal.pone.0188929

**Published:** 2017-12-11

**Authors:** István Sulykos, Zsófia Anna Gaál, István Czigler

**Affiliations:** 1 Institute of Cognitive Neuroscience and Psychology, Centre for Natural Sciences, HAS, Budapest, Hungary; 2 Eötvös Loránd University, Budapest, Hungary; Universidad de Salamanca, SPAIN

## Abstract

We investigated visual mismatch negativity (vMMN) to vanishing parts of continuously present objects by comparing the event-related potentials (ERPs) to infrequently (deviant) and frequently (standard) disappearing parts of the objects. This paradigm both excludes low-level stimulus-specific adaptation differences between the responses to deviants and standards, and increases the ecological validity of the stimuli. In comparison to frequently disappearing parts of the stimulus objects, infrequently vanishing parts elicited posterior negative event-related brain activity (vMMN). However, no vMMN emerged to the reappearance of the same parts of the objects. We compared the ERPs of an older and a younger sample of participants. In the 120–180 ms time period vMMN was similar in the two age groups, but in the 180–220 ms time period vMMN emerged only in the younger participants. We consider this difference as an index of more elaborate automatic processing of infrequent stimulus changes in younger adults.

## Introduction

This study has three aims, establishing a more ecologically valid paradigm, controlling low-level adaptation, and comparing automatic change detection in older and younger participants. Regarding the first aim, in event-related potential (ERP) research, including research on visual mismatch negativity (vMMN), the most frequent practice of stimulus delivery is the presentation of stimuli alternating with empty inter-stimulus fields. A few exceptions in the vMMN literature are the studies by Besle et al., 2005 [1] and Clery et al., 2012 [2], where the infrequent and frequent events were different deformations of circles to ellipses. Other studies with non-empty fields investigated visual masking effects with zero or short stimulus onset asynchronies (SOA), but even in these studies there were blank intervals between the trials (e.g., [3,4,5,6,7,8]). In further ERP research two frequently used stimulation procedures also apply the continuous presence of stimuli. The first is contrast-reversal stimulation, which is useful in many fields, but only infrequently applied in studying information processing issues (e.g., [9,10]). The second common stimulus type contains moving patterns [11,12]. In this case, the pattern remains visible but static during the inter-stimulus interval and the (standard or deviant) event is the abrupt start (of motion) of the pattern.

However, in everyday conditions the appearance of visual events preceded and followed by empty fields is unusual. (A rare example is a lightning on a cloudy night.) In a more usual scenario, objects appear or disappear among other objects, or objects are continuously present, but some characteristics of the objects change (they can move, rotate, be occluded by other objects, etc.). The visual system develops over the lifespan through continuous interaction with the real (and not laboratory) world. Object representations are the main building blocks of the visual percept; and the identification of the same object under various conditions (e.g., temporarily occluded object) is an adaptive response of the visual system to the natural environment. Similarly, the mechanism underlying vMMN (see [13] for a review) is a crucial part of the same visual system that results in object representations. Therefore, the theoretical connection between vMMN and object identification is a feasible option. Empirical evidence supports this assumption: vMMN’s sensitivity to object identification and categorization (for a review, see [14]) is frequently reported [15,16,17] in the vMMN literature; however in those studies the stimulus presentation is carried out using the traditional ERP research paradigm (appearance and disappearance of the whole object).

In the present study our aim was to take a step towards a naturalistic scenario by simulating the occlusion of objects. Objects were continuously present on the screen, but from time to time a part of the object disappeared.

The second aim was to set up a method for controlling low-level adaptation differences between the frequent and infrequent stimuli of the oddball sequences. In studies of vMMN unattended rare (deviant) stimuli are presented within sequences of unattended frequent (standard) ones, i.e., in passive oddball sequences. The deviant-related activity is considered to be an index of mismatch between an established (sensory) memory representation for the standard and the neural representation of an event different from the standard. Accordingly, vMMN is considered to be an additional activity elicited by the deviant, but not by the diminishing activity elicited by the frequently repeated standard (for reviews see [18,19,20]). However, an unavoidable problem of the oddball sequence is that the ERP *difference* between the deviant-related and standard-related activity includes the putative activity increase to the deviant *plus* the activity decrease to the standard. The latter effect is termed as refractoriness, habituation, repetition suppression, or stimulus-specific adaptation (for a discussion on terminology see [21]; we will use the term ‘stimulus-specific adaptation’ (SSA)). To separate the effects on the standard from the deviant-related effects, the most frequent method is the introduction of the equal probability control [22;23]. In this procedure, ERPs to stimuli physically identical to the oddball deviant are presented within a sequence of various stimuli. Within the sequence the probability of each stimulus type is equal to the probability of the oddball deviant. Accordingly, in the absence of the standard the stimuli of the control sequence cannot elicit mismatch-related activity. The ERP difference between the oddball deviant and the physically identical control from the equal probability sequence is called “genuine MMN”. As an inherent problem of this procedure, the to-be-compared ERPs are selected from highly different sequences. Furthermore, in many cases it is difficult to vary properly the stimuli of the control sequence (e.g., in case of female vs. male photographs, [24]; feature absence vs. feature presence in vMMN studies, [25]). Our paradigm attempts to solve the low-level adaptation issue using the opposite logic. Rather than using an unadapted comparison from an equal probability sequence, during the ‘inter-stimulus period’ the objects were continuously present; therefore all stimulus features were adapted. Consequently, larger activity elicited by a rarely vanishing (disappearing) feature could not be attributed to low-level SSA. It is important to note that deviant-related activity is superimposed on *offset* responses. Offset and onset ERPs are different from each other [26;27], but offset responses are relatively infrequently investigated. Furthermore, the offset of the vanishing feature is followed by feature onset. Accordingly, the rarely vanishing feature is followed by the *rare appearance* of the missing feature. Consequently, in this case re-appearance of the stimuli may elicit onset-related vMMN.

Offset stimuli are less salient than rapid onset stimuli [28,29,30,31]. In a paradigm investigating the effects of unattended stimuli (cf. vMMN paradigm) the use of less salient events is especially insightful. In our study, we made use of disappearing sides of diamonds as offset stimuli; one pair of opposite sides was the standard, and the other pair was the deviant stimulus.

As the third aim, in this study we investigated the automatic processing of rare stimuli in younger and older participants. In contrast to the auditory MMN (see [32] for a review), investigations of age-related changes of vMMN are relatively rare, and the results are equivocal. In older participants deviant motion direction elicited smaller motion-related vMMN [33], but the sample size of the study was small (7 younger, 5 middle-aged and 9 older participants). Tales et al. [34] presented task-irrelevant single or double bars (either as frequent or infrequent stimuli) within the sequence of a three stimulus (standard, deviant and target) oddball paradigm. Deviant-related negativity was smaller again in the older group; however, in a more recent study applying a similar method, Stothart et al. [35] did not confirm this result. Recently we [36] investigated age-related difference in temporal integration in a vMMN paradigm. In the control condition (without the requirement of integrating two consecutive stimuli) we obtained no age-related vMMN differences. Thus, two studies so far have reported age-related differences, but no such difference appeared in the other two studies.

## Materials and methods

15 older (mean age: 66.4 years, SD: 3.1 years) and 15 younger women (mean age: 22.4 years, SD: 1.6 year) participated in the study. For all participants lower field checkerboard stimulation resulted in a posterior negativity within the 100–180 ms range (C2 or N1 component). All of them had (corrected to) normal vision (at least 5/5 in version of the Snellen charts). No one reported any neurological or psychiatric diseases. Both the older and younger participants were paid for their contributions. We ruled out dementia-related differences between the age-groups; full scale Wechsler IQ (measured by the Hungarian version of WAIS-IV; [37]) of the older and younger group were 118.7 (SD: 15.3) and 97.6 (SD: 15.2), respectively. Written informed consent was obtained from the participants before the experimental procedure. The study was approved by the United Ethical Review Committee for Research in Psychology (Hungary).

**Fig 1:**
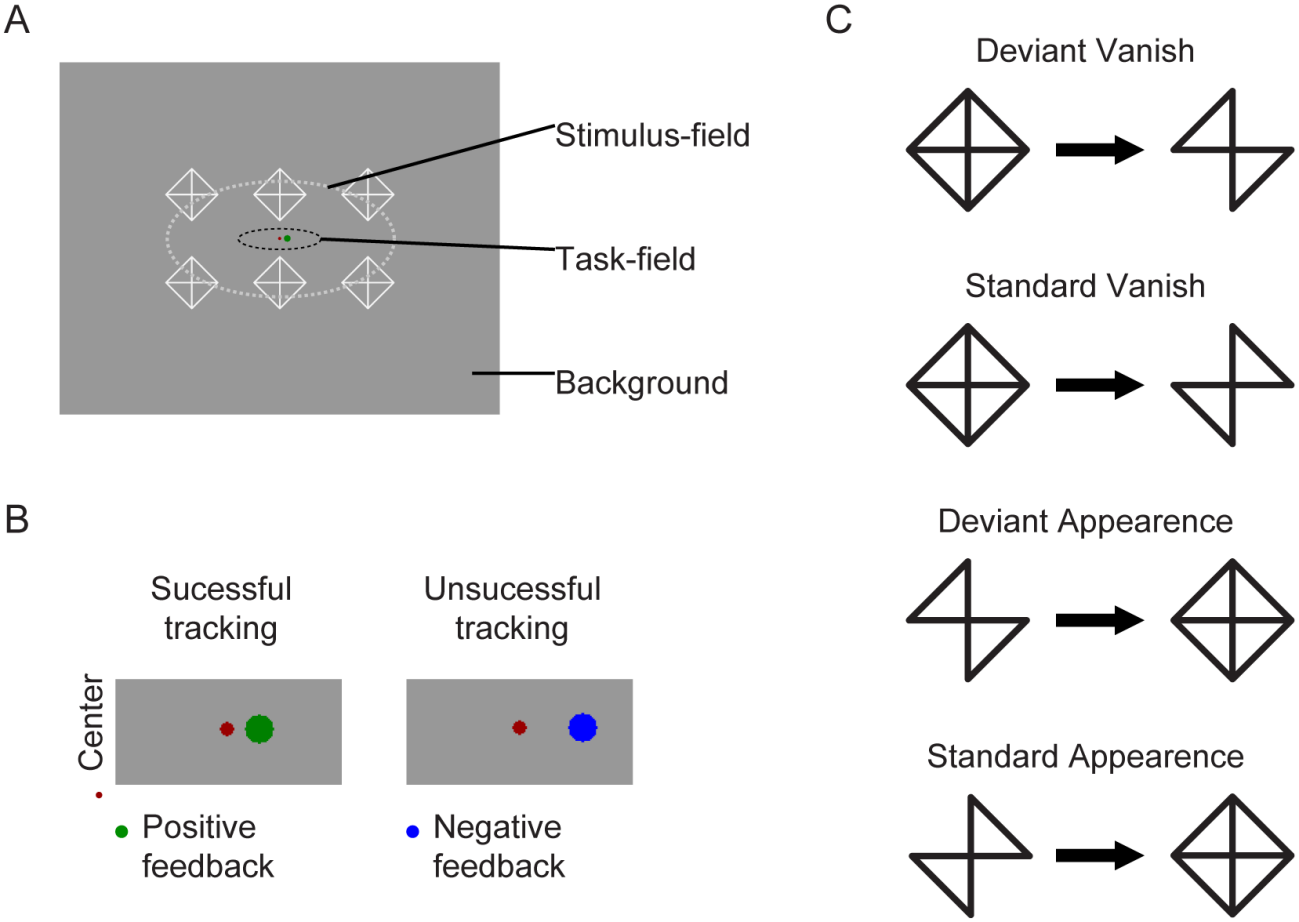
A. The stimulus display (ERP-related patterns and the task-field). B: The task field in case of successful and unsuccessful tracking. C. Sequence of stimuli; frequently vanishing elements (Standard Vanish), appearance of the pattern after the frequently vanished elements (Standard Appearance), infrequently vanished elements (Deviant Vanish), appearance of the pattern after the infrequently vanished elements (Deviant Appearance).

The experimental stimuli were presented to the participants using a 19 in. CRT monitor (Flatron 920B, 75 Hz refresh rate) from 1.4 m distance. The stimulation included both task-relevant and task-irrelevant stimuli. Fig 1 demonstrates both types of stimuli and the stimulus sequence. The task-relevant stimuli were two disks at the central area of the screen. One of the disks was red and served as a fixation point (0.11 degrees in visual angle). The second disk was green (0.22 degrees) and made horizontal pseudorandom motion around the fixation point. The task was to keep the green disk as close to the fixation point as possible with the left and right arrows of a keyboard. The error in the task was when the distance of the two disks exceeded 1.1 degrees (in case of an error, the color of the disk changed blue to provide online visual feedback). The participants’ performance (i.e. the sum of errors in one block) was reported on the screen at the end of each block.

The task-irrelevant stimuli appeared around the task-relevant stimuli. The stimuli were homogenous patterns of six identical objects (in a 2 rows by 3 columns arrangement; luminance: 101.63 cd/m^2^) against middle grey background (luminance: 36.73 cd/m^2^). There were three types of stimulus patterns. In the first one, the objects were oblique squares (similar to a diamond) with diagonals. The diameter of one diamond was 1.65 degrees; the width of the lines was 0.055 degrees. Objects of the second and third patterns were similar to the first one except that the two opposite, parallel sides (45 or 135 degree) of the square were absent (together with the diagonals, the remaining objects were similar to oblique bow ties).

The stimulus sequences were as follows. Every odd stimulus was a “diamond” pattern and every even stimulus was a “bow tie” pattern. It is important to mention that the screen was never blank. Bow ties and diamonds alternated with each other without inter-stimulus intervals. The two bow tie patterns (left and right tilted; the disappearing stimulus features were the 135-degree and the 45-degree orientated line segments, respectively) were presented in an oddball sequence. The probability of the deviant was 0.2; that is, the ratio of the two bow tie patterns was 1:4 in a sequence. The stimulus sequence resulted in four different events: rare disappearance, frequent disappearance, rare appearance and frequent appearance of stimulus feature. In a block there were 95 diamonds, 76 left tilted bow ties, and 19 right tilted bow ties. Based on the reverse control principle, we introduced an additional sequence, where the number of bow ties were interchanged (i.e. both bow tie patterns were either standard or deviant in separate sequences). There were 6 oddball and 6 reverse blocks, which resulted in 570 stimuli in each condition. The number of deviant stimuli was 114 per condition. The stimulus duration of all three patterns was 520 ms (with +/- 40 ms jitter in 13.3 ms steps).

EEG was recorded with Neuroscan recording system (Synamps2 amplifier, EasyCap, Ag/AgCl electrodes, DC-200 HZ, sampling rate: 1000 Hz). We used 38 electrode locations in accordance with the extended 10–20 system. The ground electrode was attached to the forehead. The common reference electrode was on the nose tip. Both HEOG and VEOG were recorded with bipolar configuration between two electrodes (placed lateral to the outer canthi of the two eyes or above and below the left eye, respectively).

The EEG signal was bandpass filtered offline with a non-causal Kaiser-windowed Finite Impulse Response filter (low pass filter parameters: 30 Hz of cutoff frequency, beta of 12.2653, a transition bandwidth of 10 Hz; high pass filter parameters: 0.1 Hz of cut off frequency, beta of 5.6533, a transition bandwidth of 0.2 Hz). Epochs of 500 ms (including 100 ms pre-stimulus interval serving as baseline) were extracted for all deviants and for those standards that immediately preceded the deviants. Epochs with larger than 100 μV voltage change were considered artifacts and rejected from the further processing. ERPs were calculated by averaging the extracted (and residual) epochs. According to the reverse control principle, epochs from both experimental (oddball and reverse) sequences were entered into the averaging process.

Behavioral data were defined as the total time that the ball left the target area. Age-related differences were compared using a Mann-Whitney U test.

VMMN was expected over the posterior locations within the 100–300 ms latency range. Accordingly, at the first step we calculated t-tests over this range at O1, Oz and O2 electrodes for the deviant *minus* standard difference potentials (difference from zero). Emergence of vMMN was considered if at least 10 consecutive t-values were significant (p<0.05 or less) at least over two locations. In the second step we divided the range of the ~ 100 ms duration of the deviant-related negativity into five epochs of 20 ms each, and the average of these epochs were compared in ANOVA with factors of Age (older, younger) and Location (O1, Oz, O2).

When appropriate, Greenhouse-Geisser calculation was applied. Effect size was calculated as partial eta square (η_p_^2^). As post-hoc tests Tukey HSD was applied.

## Results

Tracking performance was worse in the older group compared to the younger adults, the group averages were 13.4 s and 2.9 s, respectively. According to the Mann-Whitney test, the difference was significant, U = 8, p<0.001.

**Fig 2:**
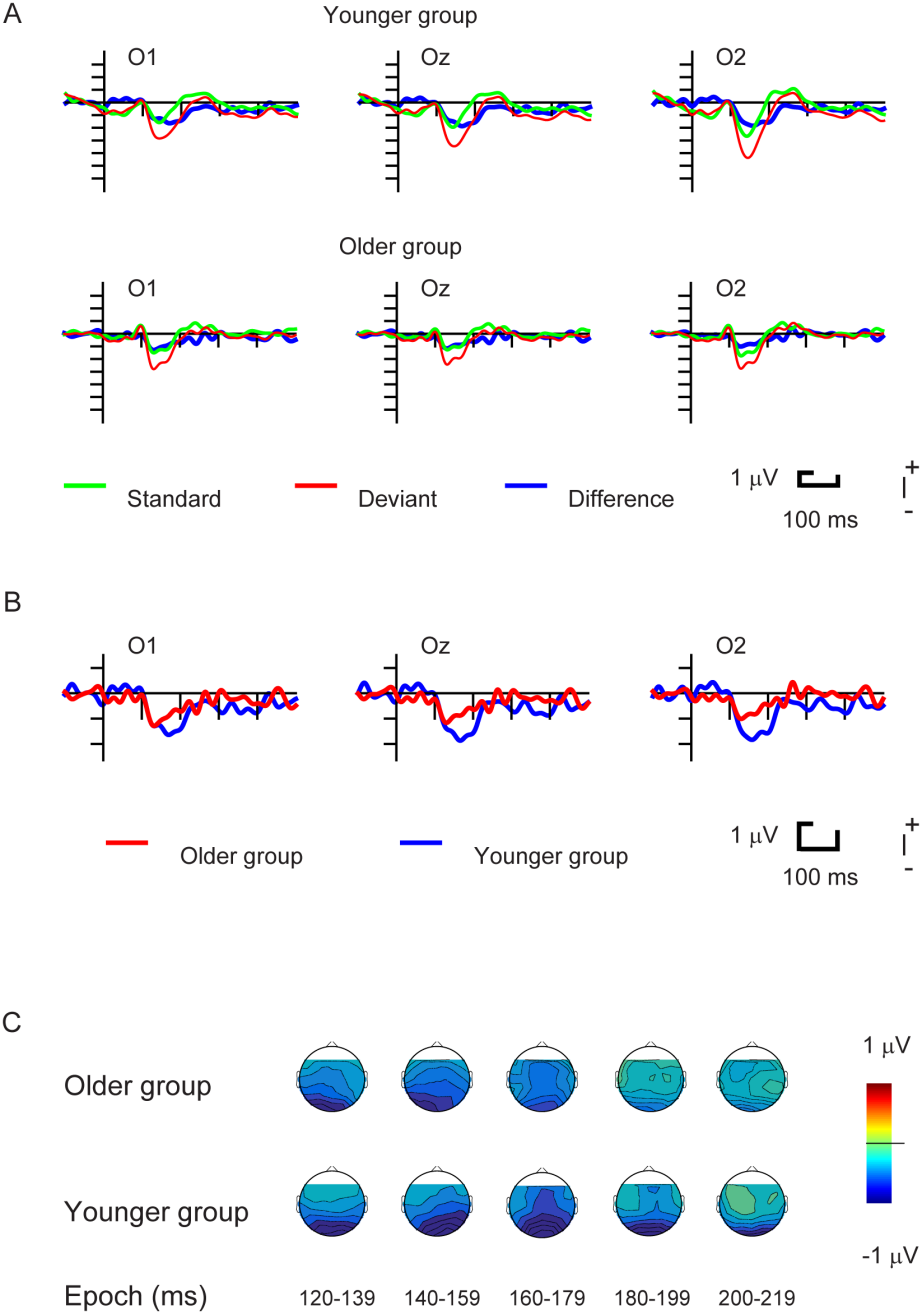
A. ERPs to Standard Vanish, Deviant Vanish and the Deviant minus Standard difference potentials in Younger and Older Group. B: Difference potentials in the two age groups. C: Surface distributions of the two age groups in the 120–219 ms latency range.

**Fig 3:**
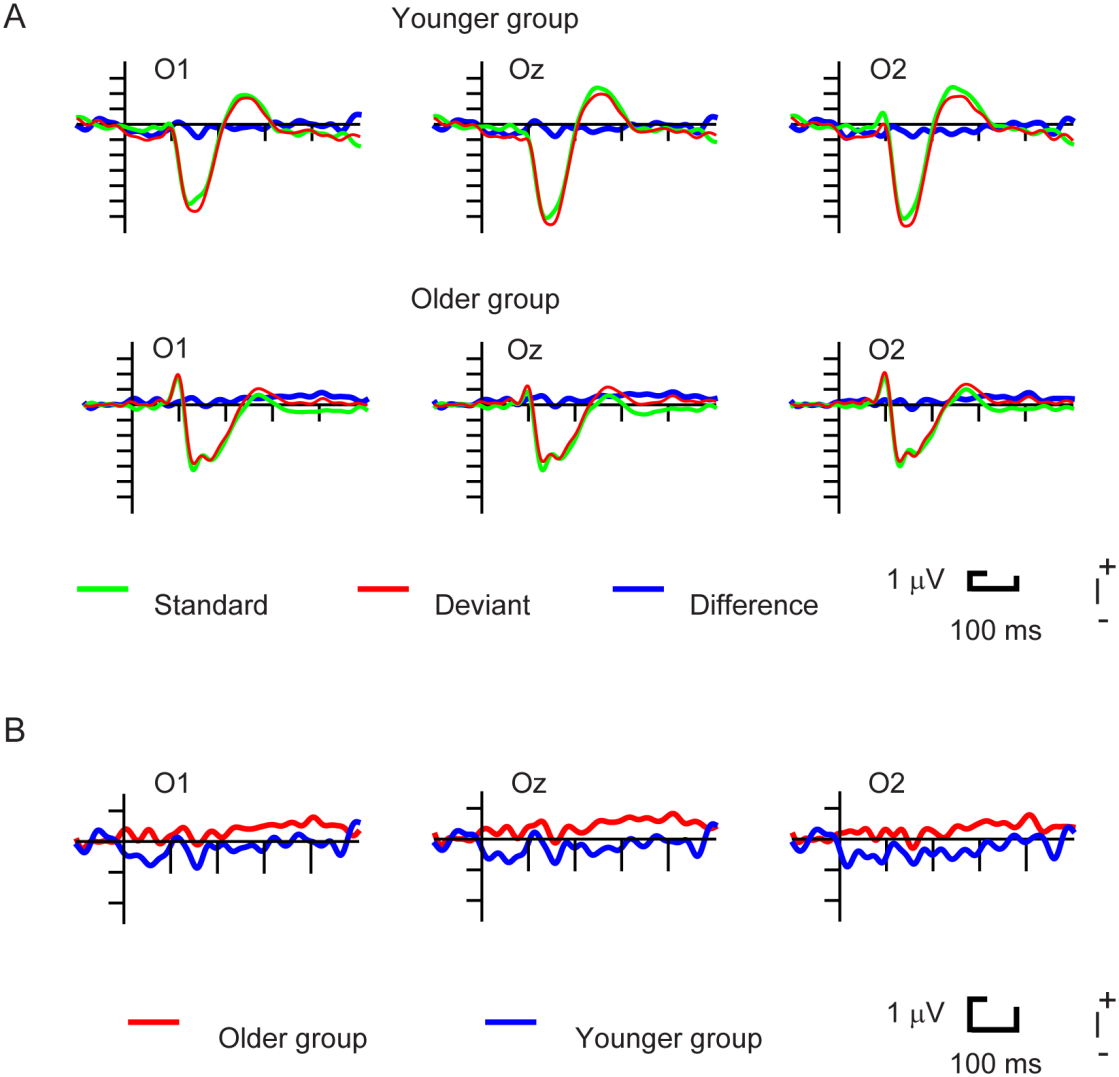
A. ERPs to Standard Appearance, Deviant Appearance and the Deviant minus Standard difference potentials in Younger and Older Group. B: Difference potentials in the two age groups.

As Fig 2 shows, deviant vanishing stimuli elicited a posterior negative wave, the vMMN. As Fig 3 shows, no such negativity emerged to the stimulus onset.

In the younger group the vanish-related difference potentials were significant (t-tests, at least 10 consecutive significant values) in the 120–220 ms range at the Oz and O2 locations, and in the 120–202 ms range at the O1 locations. In the older group this range was 116–173 ms at the three locations. For the onset stimuli we obtained no significant range. Next, we segmented the significant range (i.e., vanishing stimuli) into five time ranges within the 120–219 ms period. Then we analyzed the difference potentials in the five ranges with six separate ANOVAs (one ANOVA for the whole time range and five ANOVAs for the five consecutive epochs) with the factors of Age and Location. Table 1 contains the descriptive statistics of the five consecutive mean epochs. Fig 2 shows the scalp distributions of the vMMNs in the corresponding epochs.

**Table 1:** Mean amplitudes of the vMMN in the five consecutive ranges. The values are given in microvolts. SEM is in parenthesis. (The mean amplitudes of every participant are listed in S1 File).

Age	Location	Epochs				
		120–139	140–159	160–179	180–199	200–219
Younger	O1	-1.23(0.25)	-1.33(0.36)	-1.61(0.43)	-1.50(0.30)	-1.15(0.27)
	Oz	-1.33(0.27)	-1.61(0.34)	-1.83(0.42)	-1.65(0.28)	-1.24(0.23)
	O2	-1.43(0.30)	-1.77(0.36)	-1.71(0.46)	-1.55(0.32)	-1.29(0.25)
Older	O1	-1.22(0.25)	-1.08(0.36)	-0.88(0.43)	-0.68(0.30)	-0.58(0.27)
	Oz	-1.11(0.27)	0.93(0.34)	-0.84(0.42)	-0.62(0.28)	-0.55(0.23)
	O2	-0.97(0.30)	-0.84(0.36)	-0.78(0.46)	-0.47(0.32)	-0.39(0.25)

In a two-way ANOVA with factors of Age and Location we compared the amplitude of the difference potentials of the two groups for the whole 120–219 ms time period. The Age x Location interaction was significant, F(2,56) = 4.54, p<0.05, η_p_^2^ = 0.14, ε = 0.76. The post hoc Tukey HSD test did not indicate significant differences, but according to the results of separate t-tests at the three locations the difference was significant at O2, t(28) = 2.11, p<0.05, and approached significance at Oz (t(28) = 2.04, p = 0.05).

Fig 2 shows the surface distribution of the difference potentials in the five time ranges in the younger and older groups. As the figure shows, in the three earlier ranges the data were fairly similar, but in the older group there was hardly any negative difference in the two later ranges. Results of the ANOVAs confirmed this observation. As for the first three epochs the only significant effect appears as an Age x Location interaction in the 140–159 ms range, F(2,56) = 7.01, p<0.01, η_p_^2^ = 0.20, ε = 0.67. According to the Tukey HSD test, in the younger group the negativity was larger at O2 than at O1. In contrast with the earlier ranges, in the 180–199 and 200–219 ranges the main effect of Age was significant, F(1,28) = 5.58, p<0.05, η_p_^2^ = 0.17 and F(1,28) = 4.50, p<0.05, η_p_^2^ = 0.14, respectively.

## Discussion

In our study we introduced a new approach to investigate visual mismatch negativity (vMMN). The objects were continuously present, and stimulus presentation consisted of a part of the object disappearing repeatedly. This method has higher ecological validity compared to the usual paradigms. The main advantage of this technique is that it controls for low-level adaptation, and hence separates the standard- and deviant-related effects.

As an important aspect of the present study, in comparison to frequently disappearing features of continuously presented objects, infrequently disappearing features elicited an additional posterior negativity, the vMMN component. In contrast, there was no ERP difference between the frequently and infrequently reappearing features. This result shows that low-level stimulus-specific adaptation (SSA) or refractoriness of elementary features does not necessarily contribute to the ERP difference elicited by frequent (standard) and infrequent (deviant) events. This is because in this paradigm the absence of well adapted stimulus features elicited vMMN, but the onset of a less adapted feature did not. In this respect the present paradigm is an alternative to the equal probability paradigm [22;23] as a control for SSA, at least on the level of elementary (low level) stimulus features.

The results reveal a new aspect of the vMMN’s nature. The prevalent approach of the refractoriness (cf. [38]) issue claims that the early range of the deviant minus standard difference (before 200 ms) is inevitably due to the refractoriness effect. Our paradigm per definition ruled out the classical refractoriness effect. According to that logic, the difference wave should not have emerged after 200 ms post stimulus. In contrast, we obtained clear vMMNs in both age groups during the “refractoriness time window” in the vanish condition (and no vMMN in the appearance condition).

This contradiction leads us to the reevaluation of the refractoriness or low-level adaptation concept in the vMMN research. The source of vMMN is uncertain, according to localization attempts, occipital and other posterior structures seems to be involved (e.g., [39,40,41,42,43]). More importantly, the vMMN latency range is far beyond the onset time of the striate cortex (i.e. the location of the first entry to the visual cortex). When presented with complex visual stimuli even the temporal cortex is active within 100 ms [44]. Accordingly, vMMN activity of lower level visual areas, if such activity is present, results from feedback processes, in other words, it is a consequence of reentrant activity. Furthermore, even at the level of basic visual processing there is evidence of higher-level organization, evidenced by sensitivity to Gestalt-like grouping [45,46]. Therefore, integrated visual representations may contribute to the emergence of the negativity. According to this interpretation vMMN emerged to the irregular disappearance of visual elements (deviants), but the reappearance of the elements (irrespective of the deviant or standard one) does not violate any rule, because in both cases (standard and deviant), it is the regular object. The nature of the experimental stimulus could be the solution to the contradiction between Kimura’s and the present results. In Kimura’s study, the event was the onset of a central bar. The relative weight of the more fundamental processes (i.e. refractoriness) is presumably lower (possibly below measurable level) during the processing of Gestalt-grouping or object formation and it is higher during the processing of a singleton. In sum, it is almost impossible to match up the results between vMMNs elicited by fundamentally different stimulus types.

Furthermore, the present results corroborate the appearance of object-related vMMN [47,48,49], and vMMN to infrequent vs. frequent changes of visual objects [1,2]. It should be noted that the Besle et al. [1] and Clery et al. [2] studies–in which continuous stimulus presentation was applied–did not control low-level adaptation, because both the standard and deviant features were different from the features of the inter-stimulus field (i.e. ellipses from circles).

It is important to recognize the limits of the present paradigm. Most importantly, both the standard and deviant vanishing features were location-specific (45° lines were always at the top left and bottom right sides of the rectangles, and so were the 135° lines). Therefore it is possible that the vMMN of the present study is a sign of the sensitivity to change in frequent *versus* infrequent locations. This is a testable possibility for further research, and with the exception of a single study with highly different methods [50] has not been investigated. vMMN to change in unusual location would be a notable result in the field. Furthermore, on a theoretical level the present results do not provide direct evidence of a mechanism underlying deviant-related negativity, particularly whether it is a predictive coding process (e.g., [51,52,20]). As a conservative interpretation, processing of the infrequent vanishing element was different from the processing of frequent ones.

The other topic of the present study was the investigation of age-related aspects of automatic change detection. The results were straightforward. In the tracking task the younger group outperformed the older one. This is an expected result for visuomotor tasks requiring fast reactions (e.g., [53]). In the younger group vMMN terminated later than in the older group (~220 ms vs. ~180 ms). In the 120–180 ms time period the amplitude of the difference potential was similar in the two age groups, and in this respect the results were similar to the Stothart et al. [35] and Gaál et al. [36] studies. Comparing the whole (120–219 ms) time period, at the right occipital location the amplitude was larger in the younger group, and this result corresponded to the results reported by Tales et al. [34] and Lorenzo-López et al. [33]. However, due to the different offset of the difference potentials in the two age groups, the whole period amplitude calculation is insufficient. As Fig 2 in the study of Lorenzo-López et al. [33] shows, vMMN in the younger and middle aged groups were longer. Comparing the ERPs to deviant and standard, one cannot ignore the duration difference as Tales et al. did ([34]; see their Fig 2).

The age-related difference in the earlier vs. later time period requires interpretation, even if the possible explanations are *post hoc*. Maintaining that the posterior negativity is an indicator of different processing of the infrequent and frequent events, the process is longer in the younger group. It is unlikely that the reason of the duration difference is the diminished processing speed in the younger group, because this possibility contradicts one of the most frequent observation of aging research, that of age-related cognitive slowing (e.g., [54]). Another possibility is the longer persistence of neural activity in the younger group. However, studies on visual masking [55,56,57,58] and perceptual integration [56;59;60,36] provided opposite results, i.e., higher persistence in elderly. We hypothesize that the longer negativity is an indicator of more extensive processing. Registration of an unusual event (“there is something unusual”) is supposed to be similar in the two age groups, but the registration of change may be followed by the identification of the particular change (“what is the new event?”). This level of processing emerges more frequently in the younger group. The description fits within the predictive coding framework (e.g., [52,61,20]. In this framework mismatch components are considered error signals and are related to subsequent re-adjustment of mental representations. However, it is important to emphasize that the present results do not provide direct evidence for the operation of a predictive memory mechanism.

In conclusion, infrequent disappearance, i.e., the associated change in the shape of objects elicits vMMN, but the reappearance of the object (even if it is an infrequent change at the level of the appearance of individual features) does not. Detection of infrequent change elicits more extended activity in younger participants, and the longer activity is considered to be an indicator of more profound automatic processing.

## Supporting information

S1 File Mean amplitudes of every participant (rows) measured at every time range in every electrode location. The values are given in microvolts.
(XLSX)
